# Prevalence, Antimicrobial Resistance, and Molecular Characterization of *Campylobacter* Isolated from Broilers and Broiler Meat Raised without Antibiotics

**DOI:** 10.1128/spectrum.00251-22

**Published:** 2022-05-10

**Authors:** Sabin Poudel, Tianmin Li, Saijuan Chen, Xue Zhang, Wen-Hsing Cheng, Anuraj T. Sukumaran, Aaron S. Kiess, Li Zhang

**Affiliations:** a Department of Poultry Science, Mississippi State University, Mississippi State, Mississippi, USA; b Department of Food Science, Nutrition, and Health Promotion, Mississippi State University, Mississippi State, Mississippi, USA; c Department of Biomedical Sciences, City University of Hong Kong, Kowloon, Hong Kong, China; d Mountainous Area Research Institute of Hebei Province, Hebei Agricultural University, Baoding, Hebei, China; e Agricultural Technology Innovation Center in Mountainous Areas of Hebei Province, Baoding, Hebei, China; f Prestage Department of Poultry Science, North Carolina State University, Raleigh, North Carolina, USA; University of Adelaide

**Keywords:** *Campylobacter*, food safety, antibiotic resistance, virulence gene, no antibiotics ever

## Abstract

*Campylobacter* is one of the main bacterial pathogens that cause campylobacteriosis in the United States. Poultry is considered a major reservoir for the transmission of *Campylobacter* to humans. This study aimed to determine the prevalence and molecular characteristics of *Campylobacter* in the no-antibiotics-ever (NAE) broilers. A total of 414 samples were collected, among which 160 retail chicken samples were purchased from grocery stores and 254 samples were collected from broiler farms located in Mississippi State. The overall prevalence of *Campylobacter* was 25.4%, and a significantly higher prevalence was observed in retail chicken than in the farm samples (36.3% versus 18.5%; *P < *0.0001), respectively. The prevalence of *Campylobacter* was not different (*P = *0.263) between conventional retail (40.0%) and NAE (31.4%) retail chicken. Campylobacter jejuni was the predominant species among the positive isolates, accounting for 78.1%. Among the 82 C. jejuni isolates, 52.4% of the isolates carried the *gyrA* gene followed by the *tet*(O) gene (14.6%), whereas toxin-producing genes *cdtA*, *cdtB*, and *cdtC* were carried by 43.9%, 46.3%, and 43.9%, respectively. However, none of these virulence genes were detected in C. jejuni isolated from litter samples. Among tested C. jejuni, 13.6% of the isolates were multidrug resistant. The highest resistance was observed against nalidixic acid (49.2%), followed by tetracycline (23.7%). Our study suggests that the prevalence of *Campylobacter* was higher in retail meat samples than in environmental samples obtained from farms, and there was no difference in *Campylobacter* prevalence among conventional and NAE retail chicken.

**IMPORTANCE** The FDA antibiotic withdrawal policy has led to a shift in the production system, from conventional antibiotics fed birds to no antibiotics ever (NAE) raised birds. However, the impact of this shift to NAE on the prevalence and characteristics of *Campylobacter* has not been studied on the farm or in retail chicken meats. The objective of this study was to determine the current prevalence of *Campylobacter* and the distribution of their antimicrobial resistance and virulence genes in NAE-raised broilers. The findings of this study will help the industry to take necessary action to develop effective mitigation strategies for reducing *Campylobacter* contamination in NAE broilers.

## INTRODUCTION

Infection with *Campylobacter* is one of the leading causes of bacterial gastroenteritis worldwide (1). The incidence rate of campylobacteriosis in the United States was 19.5 cases for every 100,000 people, and the annual estimated number of infections was 1.5 million cases (2). The economic impact caused by the disease outbreaks and hospitalizations in the United States was estimated to be $1.4 to 6.9 billion (3). Similarly, a total of 220,682 confirmed human cases of campylobacteriosis were reported by 28 European Union countries in 2019 (4). Acute gastroenteritis, abdominal cramps, vomiting, and diarrhea are the most common symptoms of *Campylobacter* infections (5). In addition, *Campylobacter* infections can be more invasive and lead to severe extragastrointestinal diseases like Guillain-Barré syndrome (6), reactive arthritis (7), and irritable bowel syndrome (8).

*Campylobacter* commonly exists in the gastrointestinal tract of food animals, including swine, cattle, and especially poultry (chicken and turkey) (9, 10). Poultry provides optimal growth conditions for the bacteria, as the physiological temperature of the birds is 42°C and *Campylobacter* proliferates maximally at a 42°C temperature (11, 12). Therefore, the gastrointestinal tract of poultry harbors a much higher abundance of *Campylobacter* and acts as an ultimate host. Most *Campylobacter* spp. colonize and proliferate in the chicken gastrointestinal tract without any clinical symptoms (13), which allows this pathogen to easily contaminate the carcass during processing, which can ultimately lead to the transmission of *Campylobacter* to humans (14). Therefore, handling and consuming contaminated raw or undercooked chicken meat is considered the primary source of human *Campylobacter* infection (15). Moreover, epidemiological studies have shown that consumption of contaminated poultry products accounted for 56.5% of human *Campylobacter* infections (16).

Supportive antimicrobial treatments are not generally required for a human *Campylobacter* infection; however, immunocompromised individuals, like patients with predisposing conditions, pregnant women, children, and the elderly, may require antibiotic therapy. During these instances, fluoroquinolones (i.e., ciprofloxacin), aminoglycoside, and macrolides (azithromycin, erythromycin) are the most prescribed drugs for human campylobacteriosis (5, 17). These antibiotics commonly used in humans are also frequently used in food animals, like poultry, in order to control bacterial infections on the farms to enhance the growth performance of the animals (18). Although debatable, extensive use of antimicrobials for growth promotion in food animals is considered one of the major contributing factors for increased antimicrobial resistance worldwide (19). A study has shown the close association between the antibiotics fed to animals and the development and spread of antimicrobial resistance (AMR) in humans (20). In order to prevent a further increase and the spread of AMR, the medically important antibiotics for humans have been prohibited from poultry diets or other farm animal diets (21–23) in the United States, and they have been banned in the European Union since 2006 (24).

The Food and Drug Administration’s (FDA) antibiotic withdrawal policy has led to a shift in the poultry production system, from conventional antibiotics-fed birds to no-antibiotics-ever (NAE)-raised birds (25, 26). Broiler production under NAE systems has increased exponentially in recent years, and it accounted for almost 50% of total broiler production in the United States in 2020 (27). However, the impact of this shift to NAE on the prevalence and characteristics of *Campylobacter* has not been studied on the farm or in retail chicken meats. Therefore, it is essential to determine the current prevalence of *Campylobacter* and the distribution of its antimicrobial resistance and virulence genes in NAE-raised broilers to develop effective mitigation strategies. The objective of the present study was to investigate the prevalence of *Campylobacter* in conventional retail chicken, NAE retail chicken, and farm samples obtained from NAE broiler houses and to identify the antimicrobial resistance and virulence genes.

## RESULTS

### Prevalence of *Campylobacter* in retail chicken and broiler farm samples.

The overall prevalence of *Campylobacter* among 414 poultry samples was 25.4% (105/414). The retail chicken samples had a significantly higher prevalence of *Campylobacter* than farm samples (36.3% versus 18.5%; *P < *0.0001) (Table 1). Although conventional retail chicken samples had a numerically higher prevalence (40.0%) than NAE-raised retail chicken samples (31.4%), the difference was not significant (*P = *0.263). Prevalence of *Campylobacter* in litter, feces, and cloacal swab samples was 13.6%, 21.6%, and 22.1%, respectively (*P = *0.250) (Table 1). Prevalence of *Campylobacter* in conventional retail carcass and giblet was 44%, and prevalence in NAE retail carcass and giblet was 57.1% and 45.7%, respectively, whereas no *Campylobacter* was detected in drumsticks samples obtained from both conventional and NAE retail chicken samples. However, there was no significant difference in the prevalence of *Campylobacter* among the carcass and giblet samples obtained from both NAE and conventionally raised broilers (Table 2).

**TABLE 1:** Prevalence of *Campylobacter* species isolates from chicken samples obtained from broilers raised in conventional or NAE system and farm samples obtained from NAE houses^*a*^

Sample source	Sample no.	Positive *Campylobacter* isolates (*n*)	Prevalence^*b*^ (%)
Retail			
NAE	70	22	31.4
Con	90	36	40.0
*P*			0.263
Farm			
Litter	103	14	13.6
Feces	74	16	21.6
Cloacal swab	77	17	22.1
*P*			0.250
Total			
Retail	160	58	36.3 A
Farm	254	47	18.5 B
*P*			<0.0001
Total	414	105	25.4

a NAE, no-antibiotics-ever-raised chicken. Con, conventionally raised chicken.

b Data were analyzed using Chi-square test. Means in cells not sharing a common letter were significantly different (*P* < 0.05).

**TABLE 2:** Recovery of *Campylobacter* isolates from chicken meat samples obtained from broilers raised in conventional or NAE systems^*a*^

Sample source	No. of samples	No. of positive isolates	Prevalence^*b*^ (%)
NAE			
Drumstick	20	0	0 B
Carcass	25	11	44.0 A
Giblet	25	11	44.0 A
*P*			0.0016
Con			
Drumstick	20	0	0 B
Carcass	35	20	57.1 A
Giblet	35	16	45.7 A
*P*			0.0001

a NAE, no-antibiotics-ever-raised chicken. Con, conventionally raised chicken.

b Data were analyzed using Chi-square test. Means not sharing a common letter were significantly different (*P* < 0.05).

### Identification of *Campylobacter* species.

A total of 105 *Campylobacter* isolates from conventional chicken samples (36 isolates), NAE chicken samples (22 isolates), and farm samples (47 isolates) (Table 3) were tested. C. jejuni was the predominant species in all three types of samples collected: 22 (61.1%) isolates from conventional chicken samples, 20 (90.9%) isolates from NAE chicken samples, 40 (85.1%) isolates from farm samples, and 82 (78.1%) isolates in total. A total of 7 (6.7%) isolates were Campylobacter lari, among which 4 (11.1%) were isolated from conventional chicken samples, none were isolated from NAE chicken samples, and 3 (13.6%) were isolated from farm samples. Only 2 (1.9%) isolates were Campylobacter volucris, and 2 (5.6%) were isolated from conventional chicken samples, whereas *C. volucris* was absent in both NAE chicken samples and farm samples. Other species accounted for 14 (13.3%) isolates in total (Table 3).

**TABLE 3:** Number and percentage of different species of *Campylobacter* isolated from chicken meat samples obtained from broilers raised in conventional or NAE^*a*^ systems and farm samples obtained from NAE houses

*Campylobacter* spp.	No. of positive isolates (%) in:			
	Retail conventional samples (*n* = 36)	Retail NAE samples (*n* = 22)	Farm samples (*n* = 47)	Total incidence (*n* = 105)
C. jejuni	22 (61.1)	20 (90.9)	40 (85.1)	82 (78.1)
C. lari	4 (11.1)	0 (0.0)	3 (6.4)	7 (6.7)
*C. volucris*	2 (5.6)	0 (0.0)	0 (0.0)	2 (1.9)
Other^*b*^	8 (22.2)	2 (9.1)	4 (8.5)	14 (13.3)

a NAE, no-antibiotics-ever-raised chicken.

b *Campylobacter* strains unidentified while performing the NCBI BLAST.

### Detection of virulence genes.

The *Campylobacter* isolated in this study were tested for the presence of 13 different virulence genes (*cadF*, *jlpA*, *pebA*, *porA*, *pldA*, *ciaB*, *cdtA*, *cdtB*, *cdtC*, *flaAB*, *flgB*, and *flhB*). The prevalence of each gene in tested *Campylobacter* isolates is summarized in Table 4. The type IV secretion system virulence factor *virB9* was absent in all 105 of the *Campylobacter* isolates. Among the C. jejuni isolates, 43 (52.4%) isolates tested positive for *flgB* and *pldA* genes. Cytolethal distending toxin gene *cdtB* and *Campylobacter* adherence factor gene *cadF* were tested positive for in 38 (46.3%) C. jejuni isolates. Flagellar motility genes *flaAB* and *flhB* were possessed by 36 (43.9%) C. jejuni isolates. Five of 7 (71.4%) C. lari isolates tested positive for *cadF*, *pldA*, *cdtA*, *cdtB*, *cdtC*, *flgB*, and *flaAB* genes, whereas none of them possessed the *virB9* gene. Although only two C. volucris isolates were isolated in this study, both isolates tested positive for two virulence genes, *pldA*, and *flaAB* (Table 4). Interestingly, the C. jejuni isolates obtained from litter did not possess any of the tested 13 virulence genes (Table 5).

**TABLE 4:** Number and percentage of virulence genes present in *Campylobacter* isolated from conventional and NAE^*a*^ chicken meat samples and NAE farm samples

Virulence genes	No. of positive isolates (%) of:				
	C. jejuni (*n* = 82)	C. lari (*n* = 7)	*C. volucris* (*n* = 2)	Other^*b*^ (*n* = 14)	Total incidence (*n* = 105)
*cadF*	38 (46.3)	5 (71.4)	0 (0.0)	7 (50.0)	50 (47.6)
*jlpA*	36 (43.9)	4 (57.1)	0 (0.0)	8 (57.1)	48 (45.7)
*pebA*	24 (29.3)	3 (42.9)	0 (0.0)	5 (35.7)	32 (30.5)
*porA*	24 (29.3)	4 (57.1)	0 (0.0)	7 (50.0)	36 (34.2)
*pldA*	43 (52.4)	5 (71.4)	2 (100)	11 (78.6)	61(58.1)
*ciaB*	33 (40.2)	6 (85.7)	0 (0.0)	8 (57.1)	47 (44.8)
*cdtA*	36 (43.9)	5 (71.4)	0 (0.0)	8 (57.1)	49 (46.6)
*cdtB*	38 (46.3)	5 (71.4)	0 (0.0)	8 (57.1)	51 (48.6)
*cdtC*	36 (43.9)	5 (71.4)	0 (0.0)	8 (57.1)	49 (46.6)
*flaAB*	36 (43.9)	5 (71.4)	2 (100)	9 (64.3)	52 (49.5)
*flgB*	43 (52.4)	5 (71.4)	0 (0.0)	11 (78.6)	61 (58.1)
*flhB*	36 (43.9)	4 (57.1)	0 (0.0)	8 (57.1)	48 (45.7)
*virB9*	0 (0.0)	0 (0.0)	0 (0.0)	0 (0.0)	0 (0.0)

a NAE, no-antibiotics-ever-raised chicken.

b *Campylobacter* strains unidentified while performing the NCBI BLAST.

**TABLE 5:** Number and percentage of virulence genes present in C. jejuni isolated from conventional and NAE^*a*^ chicken meat samples and NAE farm samples

Virulence gene	No. of positive isolates (%) in:				
	Con^*b*^ meat (*n* = 22)	NAE meat (*n* = 20)	Cloaca swab (*n* = 12)	Feces (*n* = 15)	Litter (*n* = 13)
*cadF*	16 (72.7)	16 (80.0)	2 (16.7)	4 (26.7)	0 (0.0)
*jlpA*	15 (68.2)	15 (75.0)	2 (16.7)	4 (26.7)	0 (0.0)
*pebA*	9 (40.9)	12 (60.0)	2 (16.7)	1 (6.7)	0 (0.0)
*porA*	10 (45.5)	11 (55.0)	2 (16.7)	1 (6.7)	0 (0.0)
*pldA*	21 (95.5)	17 (85.0)	2 (16.7)	3 (20.0)	0 (0.0)
*ciaB*	16 (72.7)	11 (55.0)	2 (16.7)	4 (26.7)	0 (0.0)
*cdtA*	16 (72.7)	14 (70.0)	2 (16.7)	4 (26.7)	0 (0.0)
*cdtB*	18 (81.8)	14 (70.0)	2 (16.7)	4 (26.7)	0 (0.0)
*cdtC*	16 (72.7)	14 (70.0)	2 (16.7)	4 (26.7)	0 (0.0)
*flaAB*	16 (72.7)	16 (80.0)	2 (16.7)	2 (13.3)	0 (0.0)
*flgB*	21 (95.5)	17 (85.0)	2 (16.7)	3 (20.0)	0 (0.0)
*flhB*	15 (68.2)	15 (75.0)	2 (16.7)	4 (26.7)	0 (0.0)
*virB9*	0 (0.0)	0 (0.0)	0 (0.0)	0 (0.0)	0 (0.0)

a NAE, no-antibiotics-ever-raised chicken.

b Con, conventionally raised chicken.

### Detection of ARGs.

The presence of seven antibiotics resistance genes [ARGs; *aph(3′)-IIIa*, *aph(2′′)-Ig*, *bla*_OXA-61_, *bla*_OXA-184_, *tet*(O), *gyrA*, and *ermB*] were evaluated in this study; the results are shown in Tables 6 and 7. Two resistance genes, *aph(2′′)-Ig* and *ermB*, were not present in any of the *Campylobacter* isolates. The *gyrA* gene, which confers fluoroquinolones resistance, was the most prevalent resistance gene and was detected in 43 (52.43%) C. jejuni isolates, 7 (100%) C. lari isolates, and 2 (100%) *C. volucris* isolates. The tetracycline resistance gene *tet*(O) was the second most prevalent resistance gene detected and was present in 12 (14.6%) C. jejuni isolates, and a total of 17 (16.2%) isolates possessed *tet*(O) gene out of 105 total *Campylobacter* isolates. The gene *aph(3*′*)-IIIa* was present in 9 (11.0%) C. jejuni isolates and absent in C. lari and *C. volucris*. The β-lactamase resistance gene *bla*_OXA-184_ was found in 3 (2.9%) C. jejuni isolates and was absent in C. lari and *C. volucris*. Another β-lactamase resistance gene, *bla*_OXA-61_, was found in all three *Campylobacter* spp., 7 (8.5%) C. jejuni isolates, 1 (14.2%) C. lari isolate, 1 (50%) *C. volucris* isolate, and 14 (13.3%) of all *Campylobacter* isolates (Table 6). Among the C. jejuni isolates, *gyrA* resistance was observed to be higher in isolates obtained from NAE chicken samples than in those from conventional chicken samples (95.0% versus 81.8%); the lowest *gyrA* resistance was observed in the cloacal swab, 8.3% (Table 7).

**TABLE 6:** Number and percentage of antibiotic resistance genes (ARGs) of *Campylobacter* species isolated from chicken meat samples obtained from broiler raised in conventional or NAE^*a*^ system and farm samples obtained from NAE house

Antibiotic resistance genes	Expected resistance	No. of positive isolates (%) of:				
		C. jejuni (*n* = 82)	C. lari (*n* = 7)	*C. volucris* (*n* = 2)	Other^*b*^ (*n* = 14)	Total (*n* = 105)
*aph(3′)-IIIa*	Aminoglycoside	9 (11.0)	0 (0.0)	0 (0.0)	2 (14.2)	11 (10.5)
*aph(2′′)-Ig*	Aminoglycoside	0 (0.0)	0 (0.0)	0 (0.0)	0 (0.0)	0 (0.0)
*bla*_OXA-184_	β-Lactamases	3 (3.7)	0 (0.0)	0 (0.0)	0 (0.0)	3 (2.8)
*bla*_OXA-61_	β-Lactamases	7 (8.5)	1 (14.2)	1 (50.0)	5 (35.7)	14 (13.3)
*tet*(O)	Tetracycline	12 (14.6)	0 (0.0)	0 (0.0)	5 (35.7)	17 (16.2)
*gyrA*	Fluoroquinolones	43 (52.4)	7 (100)	2 (100)	10 (71.4)	62 (59.0)
*ermB*	Erythromycin	0 (0.0)	0 (0.0)	0 (0.0)	0 (0.0)	0 (0.0)

a NAE, no-antibiotics-ever-raised chicken.

b *Campylobacter* strains unidentified while performing the NCBI BLAST.

**TABLE 7:** Number and percentage of antibiotics resistance genes (ARGs) of C. jejuni isolates from chicken meat samples obtained from broilers raised in conventional or NAE^*a*^ systems and farm samples obtained from NAE houses

Antibiotic resistance genes	Expected resistance	No. of positive isolates (%) in:				
		Con^*b*^ meat (*n* = 22)	NAE meat (*n* = 20)	Cloacal swab (*n* = 12)	Feces (*n* = 15)	Litter (*n* = 13)
*aph(3′)-IIIa*	Aminoglycoside	9 (40.9)	0 (0.0)	0 (0.0)	0 (0.0)	0 (0.0)
*aph(2′′)-Ig*	Aminoglycoside	0 (0.0)	0 (0.0)	0 (0.0)	0 (0.0)	0 (0.0)
*bla*_OXA-184_	β-Lactamases	1 (4.5)	2 (10.0)	0 (0.0)	0 (0.0)	0 (0.0)
*bla*_OXA-61_	β-Lactamases	1 (4.5)	6 (30.0)	0 (0.0)	0 (0.0)	0 (0.0)
*tet*(O)	Tetracycline	6 (27.2)	6 (30.0)	0 (0.0)	0 (0.0)	0 (0.0)
*gyrA*	Fluoroquinolones	18 (81.8)	19 (95.0)	1 (8.3)	3 (20.0)	2 (15.3)
*ermB*	Erythromycin	0 (0.0)	0 (0.0)	0 (0.0)	0 (0.0)	0 (0.0)

a NAE, no-antibiotics-ever raised chicken.

b Con, conventionally raised chicken.

### Phenotypic antimicrobial susceptibility testing.

Isolates of C. jejuni (*n* = 59), C. lari (*n* = 7), and *C. volucris* (*n* = 2) were tested for phenotypic antimicrobial resistance, and the results are shown in Tables 8 and 9. Among them, 9 (13.6%) C. jejuni isolates and 2 (28.6%) C. lari isolates were multidrug-resistant (MDR) strains. Overall, 57.6%, 71.4%, and 100% of C. jejuni, C. lari, and *C. volucris* isolates were resistant to at least one tested antibiotic. The results from C. jejuni showed that the highest resistance was observed for nalidixic acid (49.2%), followed by tetracycline (23.7%), clindamycin (16.9%), erythromycin (16.9%), and ciprofloxacin (15.3%), and the lowest resistance was observed in gentamicin (6.8%; Table 8). The results from C. lari showed that the highest resistance was observed in nalidixic acid (71.4%), followed by ciprofloxacin (57.1%) and tetracycline (57.1%), and the lowest resistance was observed in erythromycin, azithromycin, and gentamicin (14.3%), respectively. Of two *C. volucris* isolates, one was resistant to ciprofloxacin and tetracycline (Table 8). Among the C. jejuni isolates from retail chicken samples, resistance was observed to be higher in conventional samples than in NAE samples (nalidixic acid, 47.6% versus 15.0%; tetracycline, 23.8% versus 10.0%; ciprofloxacin, 9.5% versus 0%), whereas azithromycin (4.8% versus 10.0%), clindamycin (9.5% versus 10.0%), and erythromycin (9.5% versus 10.0%) had higher resistance in NAE samples than in conventional chicken samples (Table 9). Among the C. jejuni isolates from farm samples, 100% of the cloacal swab and feces isolates were resistant to nalidixic acid, and 66.7% of the litter isolates were resistant to nalidixic acid (Table 9).

**TABLE 8:** Number and percentage of isolates with phenotypic antibiotics resistance (AST) of *Campylobacter* spp. isolated from chicken meat samples obtained from broilers raised in conventional or NAE systems and farm samples obtained from NAE houses^*a*^

Antibiotics	No. of positive isolates (%) of:														
	C. jejuni (*n* = 59)			C. lari (*n* = 7)			*C. volucris* (*n* = 2)			Other (*n* = 11)			Total (*n* = 79)		
	R^*b*^	I^*c*^	S^*d*^	R^*b*^	I^*c*^	S^*d*^	R^*b*^	I^*c*^	S^*d*^	R^*b*^	I^*c*^	S^*d*^	R^*b*^	I^*c*^	S^*d*^
Azithromycin	8(11.9)	1(1.7)	50(84.7)	1(14.3)	0(0.0)	6(85.7)	0(0.0)	0(0.0)	2(100)	2(18.2)	0(0.0)	9(81.8)	11(13.9)	1(1.3)	67(84.8)
Ciprofloxacin	9(15.3)	9(15.3)	41(69.5)	4(57.1)	0(0.0)	3(42.9)	1(50.0)	1(50.0)	0(0.0)	5(45.5)	0(0.0)	6(54.5)	19(24.1)	10(13.0)	50(63.3)
Clindamycin	10(16.9)	10(16.9)	39(66.1)	3(42.9)	2(28.6)	2(28.6)	0(0.0)	1(50.0)	1(50.0)	3(27.3)	2(18.2)	6(54.5)	16(20.3)	15(19.0)	48(60.8)
Erythromycin	10(16.9)	2(3.4)	47(79.7)	1(14.3)	1(14.3)	5(71.4)	0(0.0)	1(50.0)	1(50.0)	2(18.2)	1(9.1)	8(72.7)	13(16.5)	5(6.3)	61(77.2)
Gentamicin	4(6.8)	1(1.7)	54(91.5)	1(14.3)	0(0.0)	6(85.7)	0(0.0)	0(0.0)	2(100)	1(9.1)	0(0.0)	10(90.9)	6(7.6)	1(1.3)	72(91.1)
Nalidixic acid	29(49.2)	0(0.0)	30(50.8)	5(71.4)	0(0.0)	2(28.6)	0(0.0)	0(0.0)	2(100)	8(72.7)	0(0.0)	3(27.3)	42(53.2)	0(0.0)	37(46.8)
Tetracycline	14(23.7)	8(13.6)	37(62.7)	4(57.1)	0(0.0)	3(42.9)	1(50.0)	0(0.0)	1(50.0)	4(36.4)	1(9.1)	6(54.5)	23(29.1)	9(11.4)	47(59.5)

a The cutoff values for the demarcation of resistance, intermediate, and susceptibility are listed in Table 12.

b R, resistance.

c I, intermediate.

d S, susceptibility.

**TABLE 9:** Number and percentage of phenotypic antibiotics resistance (AST) of C. jejuni isolated from chicken meat samples obtained from broilers raised in conventional or NAE^*a*^ systems and farm samples obtained from NAE houses

Antibiotics	No. of positive isolates (%) in:				
	Con^*b*^ meat (*n* = 21)	NAE meat (*n* = 20)	Cloacal swab (*n *= 4)	Feces (*n* = 8)	Litter (*n* = 6)
Azithromycin	1 (4.8)	2 (10.0)	0 (0.0)	3 (37.5)	1 (16.7)
Ciprofloxacin	2 (9.5)	0 (0.0)	2 (50.0)	3 (37.5)	2 (33.3)
Clindamycin	2 (9.5)	2 (10.0)	0 (0.0)	3 (37.5)	2 (33.3)
Erythromycin	2 (9.5)	2 (10.0)	0 (0.0)	3 (37.5)	2 (33.3)
Gentamicin	1 (4.8)	0 (0.0)	0 (0.0)	2 (25.0)	1 (16.7)
Nalidixic acid	10 (47.6)	3 (15.0)	4 (100)	8 (100)	4 (66.7)
Tetracycline	5 (23.8)	2 (10.0)	2 (50.0)	2 (25.0)	3 (50.0)

a NAE, no-antibiotics-ever-raised chicken.

b Con, conventionally raised chicken.

## DISCUSSION

This study evaluates the prevalence, virulence, ARGs, and antimicrobial susceptibility testing (AST) of *Campylobacter* isolates from retail NAE and conventional chicken samples and NAE broiler farm samples. The results indicated that the prevalence of *Campylobacter* varied in different meat samples and the commercial farm samples. Irrespective of NAE-raised or conventionally raised broiler, 36.3% of retail chicken samples tested positive for *Campylobacter*. The result from this study was a number of positive isolates slightly higher than that obtained in Georgia (30%) (28). Although *Campylobacter* was frequently recovered from retail chicken carcass and giblet samples, no *Campylobacter* contamination was detected in tested drumstick samples. The variation in the results from different sample types might be due to the different sample handling, sanitation, postchill decontamination, and packaging methods during processing. In contrast to our results, Kudirkienė et al. found that 45.5% of drumsticks were contaminated with *Campylobacter* (29). Finally, no significant difference in the *Campylobacter* incidence was observed among the NAE and conventional chicken samples in this study. This result indicates that removal of the antibiotics diet did not increase the prevalence of *Campylobacter* in poultry meat, which was opposite to the previous prediction (30). Cox (30) had predicted that the removal of the antibiotic (virginiamycin) from animal diets would increase the prevalence in poultry meat and ultimately lead to an increase of human infection by 40,000 annually in the United States.

The fecal and cloacal samples obtained from the poultry houses were considered the representative indicator of the farm environment (31, 32). In farm samples obtained from the NAE-raised broiler, the overall incidence of *Campylobacter* was 18.5%. This *Campylobacter* incidence was similar to previously predicted prevalence by a meta-analysis in the conventional environment samples, 15.8% (33). This result indicates that even though there was a shift in the broiler rearing system, the prevalence of *Campylobacter* had not altered between the conventional and NAE rearing systems. It is noteworthy that the prevalence of *Campylobacter* in the meat product samples was higher than that in farm samples. It indicates the possibility of cross-contamination during the processing and distribution of retail meat. This finding is also consistent with the study conducted by Rivoal et al., who reported that the large amount of *Campylobacter* distribution in the poultry product might be due to cross-contamination during processing (34).

Among 25 *Campylobacter* spp., C. jejuni is the most prevalent species causing human infections, followed by C. coli (35). In this study, C. jejuni was the predominantly isolated species, which accounted for 78.1% of total *Campylobacter* isolates. Similar to previous studies, we have demonstrated identical isolation rates of C. jejuni from poultry samples ranging from 68.7% to 91% (10, 36, 37). However, C. coli was the second predominant species in broiler flocks (10, 36) and the most prevalent species in turkey flocks (38). However, in our study, C. coli was not detected from either broiler farm or meat samples.

The presence of the virulence genes involved in adhesion (*cadF*, *jlpA*, *pebA*, *porA*, and *pldA*), invasion (*ciaB*), toxin production (*cdtA*, *cdtB*, and *cdtC*), secretion (*virB9*), and motility (*flaAB*, *flgB*, and *flhB*) were tested. The adhesion genes play a crucial role in bacterial colonization, and the latter genes assist with the invasion of the host cells by the bacteria (39, 40). The prevalence of adhesion and invasion virulence factors varied between different samples. In previous studies, Biswas et al. and Wieczorek et al. found a higher prevalence of these adhesion and invasion genes in clinical samples than in nonclinical samples (41, 42). In this study, the prevalence of adhesion and invasion virulence factors was higher in the meat samples than in the farm samples. This indicated that C. jejuni isolated from the meat samples has a higher potential for adhesion and invasion than C. jejuni isolated from the farm samples. The cytolethal distending toxin (CDT) encoded by genes *cdtA*, *cdtB*, and *cdtC* plays an important role in toxin production and helps in the pathogenesis of *Campylobacter* (43). Among these three subunits of the *cdt* gene cluster, *cdtB* is an active toxin; the other two subunits, *cdtA* and *cdtC*, help bind and deliver the toxic gene *cdtB* (44, 45). In this study, *cdt* gene clusters were abundantly present in the isolates from conventional and NAE chicken samples compared to those from farm samples. The *cdt* gene clusters were found to be higher in patients with diarrhea (96.2%) than in the patient without diarrhea (76.5%) (46). Interestingly, the C. jejuni isolated from the litter samples tested negative for these virulence genes, which suggested that C. jejuni isolated from the meat samples is potentially more pathogenic than litter samples. Furthermore, higher prevalence of the virulence genes in the retail isolates than in those from farm samples also indicates the potential for a different source of contamination in the retail isolates. The potential sources might be transport coops, rinse water, slaughter line, and handling during processing (47).

Regarding the prevalence of antimicrobial resistance phenotypes, 13.6% of C. jejuni isolates demonstrated multidrug resistance to 3 or more different classes of antimicrobials, and 57.6% of C. jejuni isolates were resistant to at least one antimicrobial tested. The number of MDR C. jejuni isolates reported in this study was higher than the result (<3%) previously reported by NARMS integrated report and the results (3.5%) reported by Mathew et al. (48, 49), which may indicate that antibiotic resistance is spreading or increasing among the *Campylobacter* in poultry. However, in a survey conducted in human patients, C. jejuni isolates showed much higher MDR 56.8% (50). Increased MDR in *Campylobacter* might cause serious public health concerns, as it may pose a severe health issue to patients, leading to increase health care costs. Among the C. jejuni isolates, phenotypic resistance was observed in 15.3% of ciprofloxacin, whereas 52.43% of C. jejuni isolates possess *gyrA* gene in their genome, which is responsible for fluoroquinolone resistance. In this study, ciprofloxacin resistance observed in C. jejuni was lower than the national average of 28% in 2017 (51). The phenotypic resistance observed in azithromycin and erythromycin was 11.9% and 16.9%, respectively, whereas 11.0% of C. jejuni isolates possess *aph(3′)-IIIa* gene, which is responsible for aminoglycoside resistance. Phenotypic tetracycline resistance was observed in 23.7% C. jejuni isolates, whereas only 14.6% possess *tet*(O) genes in their genome. One possible reason is that other genes encoding tetracycline resistance may be present in these bacteria. These results suggested that more related genes should be examined to explore the genotypic-phenotypic discrepancies between antibiotic resistance characteristics of these *Campylobacter* isolates.

### Conclusion.

The present study showed that the predominant species of *Campylobacter* present in the processed retail chicken and on the farm was C. jejuni within Mississippi State. This study highlights the field status of antibiotic resistance and virulence of *Campylobacter*. The *Campylobacter* isolated in this study showed higher resistance to azithromycin, tetracycline, and fluoroquinolone antibiotics, and these are the most common antibiotics used for the treatment of human *Campylobacter* infection. The high level of antimicrobial resistance from the isolated *Campylobacter* strains represents a public health threat due to the potential transmission to humans. The higher prevalence of the antimicrobial resistance and virulence genes in the meat samples compared to that in the farm samples indicates that the potentially pathogenic strains of C. jejuni are circulating in retail meat, suggesting that there remains a public health and food safety threat due to potential transmission to humans. The results obtained in this study provide valuable information about the prevalence, phenotypic and genotypic antibiotics resistance, and virulence genes of *Campylobacter* present on NAE farm and retail meat, which is essential for developing effective intervention methods to control *Campylobacter* on the NAE farm and reduce foodborne infections caused by *Campylobacter* species transmitted through poultry products.

## MATERIALS AND METHODS

### Sample collection.

Retail raw chicken samples (whole chicken carcass, giblets, and drumstick) were randomly collected from 10 grocery stores within the state of Mississippi between April and July 2019. A total of 70 NAE-raised chicken samples and 90 conventional chicken samples were obtained. The samples were transported in a cooler with ice to the laboratory and processed within 12 h.

A total of 254 farm samples, including litter (*n* = 103), feces (*n* = 74), and cloacal swabs (*n* = 77), were collected randomly from four commercial broiler farms under an NAE program in the state of Mississippi between April and July 2019. The ages of the broilers for the collection of samples were between 28 to 56 days. Approximately 10 g of litter and fresh feces was aseptically collected into prelabeled sterile Whirl-Pak filter bags (Nasco Whirl-Pak standard sample bags). Cloacal swabs were taken from randomly selected birds and immersed in a sterile culture tube with 5 mL of double-strength blood-free Bolton broth (2× BF-BB) (Oxoid, CM0983).

### Bacterial isolation.

The bacterial isolation was performed based on the Microbiology Laboratory Guidebook (MLG) 41.04 method provided by the United States Department of Agriculture-Food Safety and Inspection Service (USDA-FSIS) with some modifications (52, 53). The raw poultry product samples (carcass, giblets, and drumstick) were weighed, and five times the volume of buffered peptone water (BPW) was added and mixed thoroughly by hand massaging and stomaching for 60 s. For the litter and feces samples, 10 g was prepared for *Campylobacter* enrichment by adding 90 mL of BPW, and then the sample was mixed thoroughly by gently shaking for 60 s. After preenrichment, 20 mL of the homogenized mixture was transferred into a 20 mL, 2× BF-BB supplemented with Oxoid Bolton Broth Selective supplements consisting of vancomycin, cefoperazone, trimethoprim, and amphotericin B (Oxoid, SR0183E). For the cloaca swab samples, an additional 5 mL of the 2× BF-BB supplements with antibiotics was added to the initial 5 mL of BPW, making the total volume 10 mL. The cultures were incubated at 42°C for 48 h under microaerobic conditions (85% nitrogen, 10% carbon dioxide, and 5% oxygen) using Mart anaerobic jars with an Anoxomat II system (Mart Microbiology B.V., Netherlands).

After the enrichment, each sample was streaked onto duplicate *Campylobacter* selective agar media plates. C. jejuni ATCC 33560 and C. jejuni ATCC 29428 were used as positive controls. The *Campylobacter* selective agar contained *Campylobacter* agar base (Oxoid, CM0689), selective supplements (Oxoid, SR0204E), and 5% laked horse blood (Remel, R54072). The agar plates were then incubated under the microaerophilic condition at 42°C for 48 h. All agar plates were subsequently checked for the presence of typical colonies (small to medium, grayish in coloration with an irregular or round edge, and mucoid appearance) of *Campylobacter*. One completely independent single colony was selected per sample, and the suspected colony was subcultured twice on the *Campylobacter* selective agar to obtain the pure culture.

### Identification of *Campylobacter*.

The confirmation was conducted via real-time PCR using *Campylobacter* genus-specific primers. Suspected *Campylobacter*-positive colonies were resuspended in nuclease-free water. As described previously, DNA was prepared using boiled lysates with some modification (54). Briefly, a sterile plastic needle was used to touch a portion of the suspect colony, which was then suspended in 100 μL of phosphate-buffered solution (1× PBS) solution and then centrifuged at 3,884 relative centrifugal force (rcf) using VWR mini centrifuge for 2 min to obtain a bacterial pellet. The supernatant was discarded, and 100 μL of nuclease-free water was added and mixed via vortexing. The bacterial suspension was boiled at 95°C for 5 min. The resulting solution was centrifuged at 3,884 rcf using VWR mini centrifuge for 2 min, and the supernatant was transferred into a new tube and served as the DNA template.

The real-time PCR assay was performed using forward primer R-campF2 (5′-CACGTGCTACAATGGCATAT-3′) and reverse primer R-campR2 (5′-GGCTTCATGCTCTCGAGTT-3′). Each reaction contained 5 μL of PowerUp SYBR green master mix (Applied Biosystems, USA), 3.5 μL of nuclease-free water, 0.25 μL of each forward and reverse primer (10 μM), and 1 μL of template DNA. Real-time PCR was performed using a Quant Studio 3 (Applied Biosystems, USA) with the following conditions: the initial denature step was 95°C for 20 s, 40 cycles at 95°C for 3 s, and 60°C for 20 s. Melting curve analysis was performed in the range of 60°C to 95°C at 0.5°C per 5-s increment to analyze the specificity of the primers.

### Identification of *Campylobacter* spp.

The 16S rRNA gene has been used to rapidly detect and identify bacterial species (55). The 16S rRNA gene was amplified from the DNA of the isolates by PCR using the universal forward primer 27F (5′-AGAGTTTGATCCTGGCTCAG-3′) and reverse primer 1492R (5′-CGGTTACCTTGTTACGACTT-3′). PCR was conducted by using GoTaq green master mix (Promega, Madison, WI), and an Eppendorf master cycler (Eppendorf, Westbury, NY) thermocycler was used under the following conditions: 2 min at 95°C and 35 cycles of 30 s denaturation at 95°C, 30 s annealing at 60°C, and 90 s for the extension at 72°C. The PCR products were purified using a PCR purification kit (GeneJet PCR purification kit, K0701) following the manufacturer’s instructions. The purified PCR products were Sanger sequenced by Eurofins Genomics (Eurofins, Louisville, KY). The DNA sequences obtained were compared to information in the GenBank database using the Basic Local Alignment Search Tool (BLAST) program available through the National Center for Biotechnology Information (NCBI) website (www.ncbi.nlm.nih.gov/BLAST). Based on the result of BLAST comparison, *Campylobacter* isolates were assigned to different species; however, if the BLAST hit showed a mixture of different species, then those strains were classified as others.

### Screening of virulence genes.

Two pentaplex (set A and B) and one triplex PCR (set C) assays were designed to test 13 virulence genes that are highly associated with the pathogenesis of *Campylobacter*. The gene name, thermocycler condition, primers used, and product size are listed in Table 10. The PCR products were visualized via gel electrophoresis using a 2% agarose gel stained with SYBR safe DNA gel stain (Invitrogen, Carlsbad, CA). The images were visualized using a Kodak Gel Logic 200 imaging system (Eastman Kodak Co., Rochester, NY).

**TABLE 10:** List of primers used and thermocycler setting^*a*^ used for the amplification of the virulence genes

Set	Target gene	Accession no.	Primer name	Sequence (5′ to 3′)	Length (nt)^*d*^	Temp (°C)	Amplicon size (bp)
A^*b*^	*ciaB*	NP_282066	ciaB.F335	GGTCTAACTTCATCAACCCTTTGC	24	62.9	658
			ciaB.R992	CTCATGCGGTGGCATTAGAATG	22	62.7	
	*cadF*	NP_282616	cadF.F20	GCATCCACTCTTCTATTATCCGC	23	62.8	543
			cadF.R562	ATTCCGTCTTAGTGATTCTTTGGC	24	61.2	
	*cdtA*	NP_281292	cdtA.F3	ATCGTACCTCTCCTTGGCG	19	62.3	440
			cdtAR442	CGGAGCAGCTTTAACGGTTTG	21	62.6	
	*cdtC*	NP_281290	cdtC.F260	GCTCCAAAGGTTCCATCTTCTAAG	24	62.9	263
			cdtC.R522	GCAACTCCTACTGGAGATTTGAAAG	25	62.9	
	*cdtB*	NP_281291	cdtB.F152	GCTTGAGTTGCGCTAGTTGG	20	62.4	180
			cdtB.R331	TGGAGGAACAGATGTAGGAGC	21	62.6	
B^*b*^	*virB9*	YP_980061	virB9.F429	AAGAACACGCTTTGCAATGGC	21	60.6	535
			virB9.R964	CGATGATCCTAGTCTCTACTGGAC	24	64.6	
	*pebA*	NP_282073	pebA.F40	GCTCTAGGTGCTTGTGTTGC	20	62.4	436
			pebA.R476	GTAGTTGCAGCTTGAGCCAC	20	62.4	
	*porA*	NP_282406	porA.F740	TCAACTGGACACTTGAAGGTGC	22	62.7	342
			porA.R1082	CCACCATATACGAAGTCAGCACC	23	64.8	
	*flhB*	NP_281526	flhB.F531	GGTTGCACAGCTTACTTGGC	20	62.4	257
			flhB.R788	ACATCCGCACCTGCAACATC	20	62.4	
	*jlpA*	NP_282133	jlpA.F998	GCACACAGGGAATCGACAGC	20	64.5	119
			jlpA.R1116	AAATGACGCTCCGCCCATTAAC	22	62.7	
C^*c*^	*flaAB*	NP_282485 (*flaA*), NP_282484 (*flaB*)	flaA.R1094	CAGTTGGAACAGGACTTGGAG	21	62.6	~1,500
			flaB.R253	GCTCATCCATAGCCTTATCAGCAG	24	64.6	
	*pldA*	Part of NC_002163	pldA.F422	GCCTATACTCAAACTTCTTGGTGG	24	60.6	499
			pldA.R940	AGTCTATAAGGCTTTCTCCATAGCC	25	62.9	
	*flgB*	NP_281712	flgB.F25	GAACTGGTCACTGGTGCTTTAGC	23	64.6	224
			flgB.R248	CTAGGATCAGGGAATTTCCAAGG	23	62.8	

a PCR thermocycler condition was initial denaturation at 95°C for 3 min followed by 35 cycles of 95°C for 30 s, 60°C for 30 s, 72°C for 90 s, and a final extension step of 72°C for 5 min.

b Antibiotics resistance genes run as a pentaplex PCR.

c Antibiotics resistance gene run as a triplex PCR.

d nt, nucleotide.

### Detection of ARGs.

*Campylobacter* isolates in this study were tested for the presence of ARGs. A pentaplex PCR was designed and used to detect ARGs for three different antibiotic classes: tetracycline [*tet*(O)], aminoglycosides [*aph(2’)-Ig*, *aph(3′′)-IIIa*], and β-lactam (*bla*_OXA-61_, *bla*_OXA-184_). For fluoroquinolone and erythromycin, *gyrA* and *ermB* genes were tested. The PCR mix (10 μL) comprised 5 μL GoTaq green master mix (Promega, Madison, WI), 3.5 μL of nuclease-free water, 0.25 μL of each forward and reverse primer (10 μM), and 1 μL of template DNA. The thermocycler condition and primers used for PCR are listed in Table 11. The PCR products were visualized via gel electrophoresis using a 2% agarose gel stained with SYBR safe DNA gel stain (Invitrogen, Carlsbad, CA). The images were visualized using a Kodak Gel Logic 200 imaging system (Eastman Kodak Co., Rochester, NY).

**TABLE 11:** List of primers used and thermocycler setting^*a*^ used for the amplification of the antimicrobial resistance genes

Set	Target genes	Accession no.	Orientation	Sequence (5′ to 3′)	Length (nt)	Temp (°C)	Amplicon size (bp)	Reference
A^*b*^	*aph (3′)-IIIa*	NG_047420	Forward	TGCACTTTGAACGGCATGATG	21	56.5	432	This study
			Reverse	TGTCATACCACTTGTCCGCC	20	57.3		This study
	*aph (2′′)-Ig*	NG_047407	Forward	GATTTACCTGCCTTGATTCCGG	22	56.0	523	This study
			Reverse	TTCGCCGAAATCTTTCCCA	19	54.6		This study
	*bla*_OXA-184_	NG_049485	Forward	GCTCTCAAGTGCCTGCTTTT	20	56.0	317	This study
			Reverse	AAATCCAACAATCCAAGCCAAA	22	53.6		This study
	*bla*_OXA-61_	NG_049801	Forward	CTTTCTCTCCCGCTTCCACT	20	56.8	203	This study
			Reverse	ACCAATTCTTCTTGCCACTTCTTT	24	55.3		This study
	*tet*(O)	NG_048260	Forward	AATATTCAGAGAAAAGGCGGCG	22	55.7	686	This study
			Reverse	GCAGCCATAAAGAACCCCCT	20	57.6		This study
B^*c*^	*gyrA*	L04566	Forward	GCTCTTGTTTTAGCTGATGCA	21	60.6	620	59
			Reverse	TTGTCGCCATACCTACAGCTA	21	58.7		59
C^*c*^	*ermB*	KC575115	Forward	GGGCATTTAACGACGAAACTGG	22	62.7	421	60
			Reverse	CTGTGGTATGGCGGGTAAGT	20	62.4		60

a PCR thermocycler condition was initial denaturation at 95°C for 3 min followed by 35 cycles of 95°C for 30 s, 55°C for 30 s, 72°C for 60 s, and a final extension step of 72°C for 5 min.

b Antibiotics resistance genes run as a pentaplex PCR.

c Antibiotics resistance gene run individually for PCR.

### AST.

Isolates were lost during the stock and reculture process, so isolates that were successfully recultured were tested for AST. C. jejuni (*n* = 59), C. lari (*n* = 7), and *C. volucris* (*n* = 2) were recultured from the −80°C stock culture, and phenotypic AST was tested using the Kirby-Bauer disc diffusion method. AST was conducted against seven antibiotics, which included azithromycin (AZI; Oxoid, CT0906B), ciprofloxacin (CIP; Oxoid, CT0425B), clindamycin (CLI; Oxoid, CT0064B), erythromycin (ERY; Oxoid, CT0020B), gentamicin (GEN; Oxoid, CT0024B), nalidixic acid (NAL; Oxoid, CT0031B), and tetracycline (TET; Oxoid, CT0054B). Briefly, the bacterial colony was suspended in Mueller-Hinton broth (MHB) and compared to a 0.5 McFarland Standard solution using spectrophotometry (Thermo Scientific Sensititre Nephelometer, CatLog: V301). The standardized 100 μL of MHB solution was transferred to 11 mL cation-adjusted MHB with lysed horse blood (Thermo Scientific, CP114-10) and streaked on Mueller-Hinton agar (MHA; Oxoid, CM0405) plates supplemented with 5% lysed horse blood. Antibiotic discs were placed onto the MHA plates and then incubated at 42°C for 24 h. After incubation, zones of inhibition were measured in millimeters for each plated antibiotic. The breakpoint zone of diameter from Clinical and Laboratory Standards Institute (56) for azithromycin, erythromycin, ciprofloxacin, and tetracycline was used as a standard to interpret the results. Due to the unavailability of the established standardized interpretive criteria for other antibiotics (clindamycin, gentamicin, and nalidixic acid) for *Campylobacter*, the standard guidelines for family *Enterobacteriaceae* as previously mentioned by Luangtongkum et al. and Schiaffino et al. were utilized for the interpretation of results (Table 12) (50, 57).

**TABLE 12:** Cutoff values used to determine the antibiotic resistance of *Campylobacter* along with the information about the concentration of the disc used

Antibiotics used	Disc content	Cutoff value of interpretive criteria group:			Reference source
		Resistance	Intermediate	Susceptible	
Azithromycin (AZI)	15 μg	≤12	13–15	≥16	56
Ciprofloxacin (CIP)	5 μg	≤20	21–23	≥24	56
Clindamycin (CLI)	2 μg	≤14	15–20	≥21	57
Erythromycin (ERY)	15 μg	≤12	13–15	≥16	56
Gentamicin (GEN)	10 μg	≤12	13–14	≥15	50
Nalidixic acid (NAL)	30 μg	≤19		≥20	50
Tetracycline (TET)	30 μg	≤22	23–25	≥26	56

### Statistical analysis.

Statistical analysis was performed using the Chi-square test in SAS v9.4 software (58). The level of significance (α) was set at 0.05. Data for phenotypic antibiotic resistance were categorized into three categories, resistant, intermediate, and susceptible, and all three categories were presented as a percentage. For analysis of multidrug-resistant (MDR) isolates, tested antibiotics were grouped together based on the antibiotic classes, and strains possessing resistance to three or more classes of antibiotics were considered MDR.
